# Supplementary material for: Optimization of ‘on farm’ hydropriming conditions in wheat: Soaking time and water volume have interactive effects on seed performance
Source: PLoS One. 2023 Jan 31;18(1):e0280962. doi: 10.1371/journal.pone.0280962 (PMC9888722; doi:10.1371/journal.pone.0280962)
Supplement: S1 Table — (DOCX) [file pone.0280962.s001.docx]

**S1 Table. Interactive effect of genotype and water volume of hydropriming on root length, seedling length, seedling biomass and seedling vigour index-I of wheat**

| **Root length (cm)** | | | |
| --- | --- | --- | --- |
| **Genotype🠪**  **Water volume🠇** | **WH 1105** | **WH 1124** | **KRL 213** |
| **Half volume** | 19.70 a | 20.93 a | 19.59 a |
| **Equal volume** | 19.30 b | 21.07 a | 19.42 ab |
| **Double volume** | 19.20 b | 21.00 a | 19.17 b |
| **Seedling length (cm)** | | | |
| **Genotype🠪**  **Water volume🠇** | **WH 1105** | **WH 1124** | **KRL 213** |
| **Half volume** | 29.71 a | 30.30 a | 29.74 a |
| **Equal volume** | 29.19 b | 30.44 a | 29.57 ab |
| **Double volume** | 28.87 b | 30.30 a | 29.21 b |
| **Seedling fresh weight (mg)** | | | |
| **Genotype🠪**  **Water volume🠇** | **WH 1105** | **WH 1124** | **KRL 213** |
| **Half volume** | 135.82 a | 184.95 a | 139.28 a |
| **Equal volume** | 136.99 a | 184.42 a | 141.06 a |
| **Double volume** | 128.19 b | 185.73 a | 139.32 a |
| **Seedling dry weight (mg)** | | | |
| **Genotype🠪**  **Water volume🠇** | **WH 1105** | **WH 1124** | **KRL 213** |
| **Half volume** | 13.80 a | 15.95 b | 13.86 a |
| **Equal volume** | 13.68 a | 16.22 a | 14.10 a |
| **Double volume** | 13.30 b | 16.27 a | 14.09 a |
| **Seedling vigour index-II** | | | |
| **Genotype🠪**  **Water volume🠇** | **WH 1105** | **WH 1124** | **KRL 213** |
| **Half volume** | 1306 a | 1501 a | 1280 a |
| **Equal volume** | 1288 ab | 1522 a | 1299 a |
| **Double volume** | 1264 b | 1520 a | 1299 a |

Values with different letters within a column (for each parameter) differ significantly from each other (P < 0.05)
