# Supplementary material for: Optimization of ‘on farm’ hydropriming conditions in wheat: Soaking time and water volume have interactive effects on seed performance
Source: PLoS One. 2023 Jan 31;18(1):e0280962. doi: 10.1371/journal.pone.0280962 (PMC9888722; doi:10.1371/journal.pone.0280962)
Supplement: S2 Table — (DOCX) [file pone.0280962.s002.docx]

**S2 Table. Interactive effect of genotype and soaking duration of hydropriming on germination characteristics, seedling growth and seedling vigour indices of wheat**

| **Standard germination (%)** | | | | | | | | |
| --- | --- | --- | --- | --- | --- | --- | --- | --- |
| **Soaking duration🠪**  **Genotype🠇** | **Control (Unprimed)** | **1 hr** | **2 hrs** | **4 hrs** | **8 hrs** | **12 hrs** | **16 hrs** | **20 hrs** |
| **WH 1105** | 94.00 a | 95.39 a | 95.94 a | 96.56 a | 97.44 a | 97.61 a | 95.17 a | 83.56 a |
| **WH 1124** | 93.67 a | 94.83 a | 95.61 ab | 96.00 ab | 96.94 a | 97.17 a | 93.50 b | 81.89 b |
| **KRL 213** | 91.00 b | 92.67 b | 94.22 b | 94.89 b | 97.06 a | 97.61 a | 89.44 c | 79.67 c |
| **Germination speed** | | | | | | | | |
| **Soaking duration🠪**  **Genotype🠇** | **Control (Unprimed)** | **1 hr** | **2 hrs** | **4 hrs** | **8 hrs** | **12 hrs** | **16 hrs** | **20 hrs** |
| **WH 1105** | 43.75 b | 54.44 ab | 61.19 ab | 69.31 a | 74.69 a | 78.03 a | 68.64 b | 56.92 ab |
| **WH 1124** | 46.50 a | 55.08 a | 62.72 a | 70.56 a | 76.33 a | 79.33 a | 70.53 a | 58.39 a |
| **KRL 213** | 41.92 b | 52.86 b | 59.47 b | 67.44 b | 72.61 b | 75.50 b | 66.56 c | 55.19 b |
| **Shoot length (cm)** | | | | | | | | |
| **Soaking duration🠪**  **Genotype🠇** | **Control (Unprimed)** | **1 hr** | **2 hrs** | **4 hrs** | **8 hrs** | **12 hrs** | **16 hrs** | **20 hrs** |
| **WH 1105** | 9.05 a | 9.67 a | 10.08 a | 9.72 b | 10.29 ab | 10.91 a | 9.81 b | 9.35 b |
| **WH 1124** | 7.68 b | 9.00 b | 9.14 b | 9.67 b | 10.03 b | 10.23 c | 9.78 b | 8.78 c |
| **KRL 213** | 9.31 a | 9.94 a | 10.10 a | 10.31 a | 10.55 a | 10.58 b | 10.43 a | 9.79 a |
| **Root length (cm)** | | | | | | | | |
| **Soaking duration🠪**  **Genotype🠇** | **Control (Unprimed)** | **1 hr** | **2 hrs** | **4 hrs** | **8 hrs** | **12 hrs** | **16 hrs** | **20 hrs** |
| **WH 1105** | 18.73 b | 19.03 b | 19.30 b | 19.77 b | 20.56 b | 20.79 b | 18.87 c | 18.13 b |
| **WH 1124** | 19.59 a | 20.46 a | 20.86 a | 21.50 a | 21.88 a | 22.25 a | 21.61 a | 20.30 a |
| **KRL 213** | 18.41 b | 19.15 b | 19.34 b | 19.70 b | 20.09 b | 20.27 b | 19.55 b | 18.62 b |
| **Seedling length (cm)** | | | | | | | | |
| **Soaking duration🠪**  **Genotype🠇** | **Control (Unprimed)** | **1 hr** | **2 hrs** | **4 hrs** | **8 hrs** | **12 hrs** | **16 hrs** | **20 hrs** |
| **WH 1105** | 27.78 a | 28.70 b | 29.38 a | 29.49 b | 30.84 b | 31.70 b | 28.68 c | 27.48 b |
| **WH 1124** | 27.28 a | 29.46 a | 30.00 a | 31.17 a | 31.91 a | 32.48 a | 31.40 a | 29.08 a |
| **KRL 213** | 27.72 a | 29.09 b | 29.35 a | 30.01 b | 30.64 b | 30.85 c | 29.98 b | 28.41 a |
| **Seedling fresh weight (mg)** | | | | | | | | |
| **Soaking duration🠪**  **Genotype🠇** | **Control (Unprimed)** | **1 hr** | **2 hrs** | **4 hrs** | **8 hrs** | **12 hrs** | **16 hrs** | **20 hrs** |
| **WH 1105** | 111.39 c | 114.69 c | 123.67 c | 136.80 b | 157.63 b | 163.43 b | 136.26 b | 125.46 b |
| **WH 1124** | 150.90 a | 175.01 a | 183.07 a | 193.70 a | 203.15 a | 204.35 a | 194.52 a | 175.56 a |
| **KRL 213** | 130.39 b | 135.13 b | 138.63 b | 142.67 b | 149.80 b | 153.61 c | 143.26 b | 128.56 b |
| **Seedling dry weight (mg)** | | | | | | | | |
| **Soaking duration🠪**  **Genotype🠇** | **Control (Unprimed)** | **1 hr** | **2 hrs** | **4 hrs** | **8 hrs** | **12 hrs** | **16 hrs** | **20 hrs** |
| **WH 1105** | 13.14 b | 13.50 b | 13.74 b | 13.80 b | 14.62 b | 14.49 b | 13.19 c | 12.22 c |
| **WH 1124** | 15.14 a | 15.80 a | 16.14 a | 16.59 a | 16.81 a | 17.02 a | 16.29 a | 15.34 a |
| **KRL 213** | 13.32 b | 13.78 b | 14.06 b | 14.28 b | 14.75 b | 14.88 b | 13.89 b | 13.17 b |
| **Seedling vigour index-I** | | | | | | | | |
| **Soaking duration🠪**  **Genotype🠇** | **Control (Unprimed)** | **1 hr** | **2 hrs** | **4 hrs** | **8 hrs** | **12 hrs** | **16 hrs** | **20 hrs** |
| **WH 1105** | 2611 a | 2738 ab | 2819 ab | 2847 b | 3005 b | 3094 ab | 2731 b | 2294 ab |
| **WH 1124** | 2555 ab | 2793 a | 2868 a | 2992 a | 3094 a | 3156 a | 2937 a | 2381 a |
| **KRL 213** | 2523 b | 2696 b | 2765 b | 2848 b | 2973 b | 3011 b | 2684 b | 2262 b |
| **Seedling vigour index-II** | | | | | | | | |
| **Soaking duration🠪**  **Genotype🠇** | **Control (Unprimed)** | **1 hr** | **2 hrs** | **4 hrs** | **8 hrs** | **12 hrs** | **16 hrs** | **20 hrs** |
| **WH 1105** | 1235 b | 1288 b | 1319 b | 1333 b | 1425 b | 1414 b | 1256 b | 1020 b |
| **WH 1124** | 1418 a | 1499 a | 1544 a | 1593 a | 1630 a | 1654 a | 1523 a | 1256 a |
| **KRL 213** | 1212 b | 1277 b | 1325 b | 1355 b | 1432 b | 1452 b | 1243 b | 1049 b |

Values with different letters within a column (for each parameter) differ significantly from each other (P < 0.05)
