# Supplementary material for: Optimization of ‘on farm’ hydropriming conditions in wheat: Soaking time and water volume have interactive effects on seed performance
Source: PLoS One. 2023 Jan 31;18(1):e0280962. doi: 10.1371/journal.pone.0280962 (PMC9888722; doi:10.1371/journal.pone.0280962)
Supplement: S3 Table — (DOCX) [file pone.0280962.s003.docx]

**S3 Table. Interactive effect of genotype and temperature on seedling growth parameters of wheat**

|  | **Shoot length (cm)** | | **Root length (cm)** | | **Seedling length (cm)** | | **Seedling fresh weight (mg)** | | **Seedling dry weight (mg)** | |
| --- | --- | --- | --- | --- | --- | --- | --- | --- | --- | --- |
| **Temperature🠪**  **Genotype🠇** | **20°C** | **25°C** | **20°C** | **25°C** | **20°C** | **25°C** | **20°C** | **25°C** | **20°C** | **25°C** |
| **WH 1105** | 8.89 a | 10.83 b | 18.42 b | 20.37 c | 27.31 b | 31.20 b | 130.46 b | 136.87 c | 13.01 b | 14.18 c |
| **WH 1124** | 8.10 b | 10.48 c | 19.97 a | 22.14 a | 28.08 a | 32.62 a | 177.04 a | 193.02 a | 14.84 a | 17.44 a |
| **KRL 213** | 8.84 a | 11.39 a | 17.76 c | 21.02 b | 26.60 c | 32.41 a | 126.07 c | 153.70 b | 12.66 c | 15.38 b |

Values with different letters within a column (for each parameter) differ significantly from each other (P < 0.05)
