# Supplementary material for: Optimization of ‘on farm’ hydropriming conditions in wheat: Soaking time and water volume have interactive effects on seed performance
Source: PLoS One. 2023 Jan 31;18(1):e0280962. doi: 10.1371/journal.pone.0280962 (PMC9888722; doi:10.1371/journal.pone.0280962)
Supplement: S4 Table — (DOCX) [file pone.0280962.s004.docx]

**S4 Table. Interactive effect of genotype and temperature on seedling vigour indices of wheat**

|  | **Seedling vigour index-I** | | **Seedling vigour index-II** | |
| --- | --- | --- | --- | --- |
| **Temperature🠪**  **Genotype🠇** | **20°C** | **25°C** | **20°C** | **25°C** |
| **WH 1105** | 2588.78 b | 2945.17 b | 1233.83 b | 1338.40 c |
| **WH 1124** | 2634.22 a | 3060.53 a | 1392.40 a | 1636.49 a |
| **KRL 213** | 2459.51 c | 2979.31 b | 1171.16 c | 1414.25 b |

Values with different letters within a column (for each parameter) differ significantly from each other (P < 0.05)
