# Supplementary material for: Optimization of ‘on farm’ hydropriming conditions in wheat: Soaking time and water volume have interactive effects on seed performance
Source: PLoS One. 2023 Jan 31;18(1):e0280962. doi: 10.1371/journal.pone.0280962 (PMC9888722; doi:10.1371/journal.pone.0280962)
Supplement: S5 Table — (DOCX) [file pone.0280962.s005.docx]

**S5 Table. Interactive effect of soaking duration and temperature on shoot length, root length, seedling length and seedling vigour index-I of wheat**

|  | **Shoot length (cm)** | | **Root length (cm)** | | **Seedling length (cm)** | | **Seedling vigour index-I** | |
| --- | --- | --- | --- | --- | --- | --- | --- | --- |
| **Temperature🠪**  **Soaking duration🠇** | **20°C** | **25°C** | **20°C** | **25°C** | **20°C** | **25°C** | **20°C** | **25°C** |
| **Control (Unprimed)** | 7.51 d | 9.85 d | 16.96 e | 20.53 cd | 24.46 d | 30.39 c | 2283 e | 2810 e |
| **1 hour** | 8.27 c | 10.80 d | 18.06 d | 21.03 bc | 26.33 c | 31.84 b | 2489 d | 2996 cd |
| **2 hours** | 8.45 c | 11.03 b | 18.43 cd | 21.24 b | 26.88 c | 32.27 b | 2565 cd | 3070 bc |
| **4 hours** | 8.87 b | 10.94 b | 19.14 b | 21.51 ab | 28.01 b | 32.44 b | 2688 b | 3102 b |
| **8 hours** | 9.07 ab | 11.51 a | 19.82 a | 21.86 a | 28.89 a | 33.37 a | 2806 a | 3242 a |
| **12 hours** | 9.36 a | 11.78 a | 20.11 a | 22.09 a | 29.48 a | 33.88 a | 2875 a | 3300 a |
| **16 hours** | 9.02 b | 10.99 b | 18.95 bc | 21.07 bc | 27.97 b | 32.07 b | 2595 c | 2973 d |
| **20 hours** | 8.33 c | 10.28 c | 17.96 | 20.07 d | 26.30 c | 30.35 c | 2156 f | 2468 f |

Values with different letters within a column (for each parameter) differ significantly from each other (P < 0.05)
