# Supplementary material for: Optimization of ‘on farm’ hydropriming conditions in wheat: Soaking time and water volume have interactive effects on seed performance
Source: PLoS One. 2023 Jan 31;18(1):e0280962. doi: 10.1371/journal.pone.0280962 (PMC9888722; doi:10.1371/journal.pone.0280962)
Supplement: S6 Table — (DOCX) [file pone.0280962.s006.docx]

**S6 Table. Interactive effect of genotype, soaking duration and temperature on shoot length, root length, seedling length and seedling vigour index-I of wheat**

|  | **Shoot length (cm)** | | | | | | | | |
| --- | --- | --- | --- | --- | --- | --- | --- | --- | --- |
| **Temperature** | **Soaking duration🠪**  **Genotype🠇** | **Control (Unprimed)** | **1 hr** | **2 hrs** | **4 hrs** | **8 hrs** | **12 hrs** | **16 hrs** | **20 hrs** |
| **20°C** | **WH 1105** | 8.16 a | 8.48 a | 8.67 a | 8.90 a | 8.91 b | 9.83 a | 9.17 a | 8.99 a |
|  | **WH 1124** | 6.47 b | 7.87 b | 8.01 b | 8.70 a | 8.89 b | 8.88 c | 8.51 b | 7.50 c |
|  | **KRL 213** | 7.89 a | 8.46 a | 8.67 a | 9.01 a | 9.40 a | 9.38 b | 9.39 a | 8.51 b |
| **25°C** | **WH 1105** | 9.93 b | 10.85 b | 11.49 a | 10.54 b | 11.66 a | 12.00 a | 10.46 c | 9.71 c |
|  | **WH 1124** | 8.90 c | 10.13 c | 10.26 b | 10.65 b | 11.17 b | 11.57 b | 11.06 b | 10.07 b |
|  | **KRL 213** | 10.73 a | 11.43 a | 11.35 a | 11.62 a | 11.69 a | 11.78 ab | 11.46 a | 11.07 a |
|  | **Root length (cm)** | | | | | | | | |
| **Temperature** | **Soaking duration🠪**  **Genotype🠇** | **Control (Unprimed)** | **1 hr** | **2 hrs** | **4 hrs** | **8 hrs** | **12 hrs** | **16 hrs** | **20 hrs** |
| **20°C** | **WH 1105** | 17.35 a | 17.76 b | 17.88 b | 18.61 b | 19.95 b | 20.31 b | 18.01 b | 17.50 b |
|  | **WH 1124** | 16.47 b | 19.07 a | 19.77 a | 20.77 a | 21.18 a | 21.41 a | 20.83 a | 19.28 a |
|  | **KRL 213** | 17.05 ab | 17.34 b | 17.63 b | 18.03 b | 18.33 c | 18.61 c | 18.01 b | 17.10 b |
| **25°C** | **WH 1105** | 20.11 b | 20.30 b | 20.72 b | 20.94 b | 21.16 c | 21.26 c | 19.72 c | 18.76 c |
|  | **WH 1124** | 21.72 a | 21.84 a | 21.95 a | 22.22 a | 22.58 a | 23.09 a | 22.39 a | 21.32 a |
|  | **KRL 213** | 19.77 b | 20.96 b | 21.04 b | 21.37 b | 21.85 b | 21.93 b | 21.09 b | 20.14 b |
|  | **Seedling length (cm)** | | | | | | | | |
| **Temperature** | **Soaking duration🠪**  **Genotype🠇** | **Control (Unprimed)** | **1 hr** | **2 hrs** | **4 hrs** | **8 hrs** | **12 hrs** | **16 hrs** | **20 hrs** |
| **20°C** | **WH 1105** | 25.51 a | 26.24 ab | 26.55 b | 27.51 b | 28.86 b | 30.14 a | 27.18 b | 26.50 a |
|  | **WH 1124** | 22.93 b | 26.94 a | 27.78 a | 29.47 a | 30.07 a | 30.30 a | 29.34 a | 26.78 a |
|  | **KRL 213** | 24.94 a | 25.80 b | 26.30 b | 27.04 b | 27.73 c | 27.99 b | 27.40 b | 25.61 b |
| **25°C** | **WH 1105** | 30.05 a | 31.16 b | 32.20 a | 31.47 b | 32.82 b | 33.26 b | 30.18 c | 28.47 b |
|  | **WH 1124** | 30.62 a | 31.97 a | 32.21 a | 32.87 a | 33.75 a | 34.66 a | 33.46 a | 31.39 a |
|  | **KRL 213** | 30.50 a | 32.38 a | 32.39 a | 32.98 a | 33.54 b | 33.71 b | 32.56 b | 31.20 a |
|  | **Seedling vigour index-I** | | | | | | | | |
| **Temperature** | **Soaking duration🠪**  **Genotype🠇** | **Control (Unprimed)** | **1 hr** | **2 hrs** | **4 hrs** | **8 hrs** | **12 hrs** | **16 hrs** | **20 hrs** |
| **20°C** | **WH 1105** | 2415 a | 2508 a | 2552 ab | 2665 b | 2819 a | 2947 a | 2593 b | 2212 a |
|  | **WH 1124** | 2141 c | 2554 a | 2652 a | 2828 a | 2913 a | 2949 a | 2742 a | 2201 a |
|  | **KRL 213** | 2293 b | 2404 b | 2490 b | 2572 b | 2687 b | 2728 b | 2450 c | 2054 b |
| **25°C** | **WH 1105** | 2804 ab | 2967 a | 3085 a | 3028 b | 3191 a | 3242 b | 2868 b | 2377 b |
|  | **WH 1124** | 2879 a | 3034 a | 3088 a | 3157 a | 3274 a | 3361 a | 3132 a | 2559 a |
|  | **KRL 213** | 2746 b | 2986 a | 3038 a | 3122 ab | 3261 a | 3296 ab | 2919 b | 2467 ab |
| Values with different letters within a column (for each temperature level) differ significantly from each other (P < 0.05) for each parameter | | | | | | | | | |
