# Supplementary material for: Optimization of ‘on farm’ hydropriming conditions in wheat: Soaking time and water volume have interactive effects on seed performance
Source: PLoS One. 2023 Jan 31;18(1):e0280962. doi: 10.1371/journal.pone.0280962 (PMC9888722; doi:10.1371/journal.pone.0280962)
Supplement: S7 Table — (DOCX) [file pone.0280962.s007.docx]

**S7 Table. Interactive effect of genotype, water volume and temperature on root length of wheat seedlings**

| **Temperature** | **Genotype🠪**  **Water volume🠇** | **WH 1105** | **WH 1124** | **KRL 213** |
| --- | --- | --- | --- | --- |
| **20°C** | **Half volume** | 18.76 a | 19.67 b | 18.04 a |
|  | **Equal volume** | 18.34 b | 19.74 ab | 17.70 ab |
|  | **Double volume** | 18.16 b | 20.14 a | 17.55 b |
| **25°C** | **Half volume** | 20.63 a | 22.19 ab | 21.14 a |
|  | **Equal volume** | 20.25 a | 22.39 a | 21.13 a |
|  | **Double volume** | 20.24 a | 21.85 b | 20.78 a |

Values with different letters within a column (for each temperature level) differ significantly from each other (P < 0.05)
