# Supplementary material for: Optimization of ‘on farm’ hydropriming conditions in wheat: Soaking time and water volume have interactive effects on seed performance
Source: PLoS One. 2023 Jan 31;18(1):e0280962. doi: 10.1371/journal.pone.0280962 (PMC9888722; doi:10.1371/journal.pone.0280962)
Supplement: S8 Table — (DOCX) [file pone.0280962.s008.docx]

**S8 Table. Analysis of Variance (F-value) for effects of drying, temperature, water volume and soaking duration on germination characteristics, seedling growth and vigour indices of the wheat genotype WH 1105**

| **Source of variation** | **DF** | **Standard germination** | **Germination speed** | **Shoot length** | **Root length** | **Seedling length** | **Seedling fresh weight** | **Seedling dry weight** | **Seedling vigour**  **index-I** | **Seedling vigour**  **index-II** |
| --- | --- | --- | --- | --- | --- | --- | --- | --- | --- | --- |
| Drying | 1 | 5.87* | 1373.88** | 77.08** | 8.60** | 46.60** | 14.92** | 3.28 | 51.72** | 7.46** |
| Temperature | 1 | 19.92** | 114.33** | 827.24** | 61.31** | 438.21** | 143.73* | 53.02** | 195.31** | 25.17** |
| Water volume | 2 | 0.30 | 18.78** | 3.36* | 4.59* | 5.91** | 0.07 | 0.29 | 5.15** | 0.27 |
| Soaking duration | 3 | 16.95** | 404.69** | 60.28** | 11.47** | 43.34** | 37.02** | 2.02 | 62.58** | 8.63** |
| Temperature × Water volume | 2 | 0.78 | 0.07 | 1.78 | 0.45* | 0.25 | 1.62 | 0.04 | 0.35 | 0.21 |
| Temperature × Duration | 3 | 4.66** | 16.39** | 1.89 | 1.90 | 2.94* | 3.72* | 2.88* | 8.62** | 2.94* |
| Volume × Duration | 6 | 0.54 | 5.13** | 0.96 | 1.95 | 2.41* | 1.05 | 0.72 | 2.02 | 0.55 |
| Drying × Temperature | 1 | 0.59 | 21.58** | 32.02** | 3.79 | 19.76** | 1.17 | 0.61 | 17.65** | 0.15 |
| Drying × Water volume | 2 | 0.27 | 1.45 | 1.89 | 0.73 | 1.81 | 4.38* | 3.52* | 1.80 | 3.83* |
| Drying × Soaking duration | 3 | 1.71 | 169.68** | 9.23** | 5.14** | 7.79** | 7.52** | 2.19 | 9.65** | 2.45 |
| Temperature × Water volume × Soaking duration | 6 | 1.10 | 1.88 | 0.78 | 0.47 | 0.45 | 1.68 | 0.62 | 0.07 | 0.28 |
| Drying × Temperature × Water volume | 2 | 0.04 | 1.77 | 1.05 | 3.69* | 2.70 | 0.68 | 1.40 | 1.52 | 1.11 |
| Drying × Temperature × Soaking duration | 3 | 2.19 | 4.83** | 5.19** | 5.36** | 5.18* | 4.51** | 0.80 | 5.43** | 0.48 |
| Drying × Water volume × Soaking duration | 6 | 1.28 | 3.12** | 0.83 | 1.18 | 0.83 | 1.74 | 0.56 | 2.00* | 0.96 |
| Drying × Temperature × Water volume × Soaking duration | 6 | 1.71 | 5.42** | 0.64 | 0.88 | 0.47 | 0.77 | 0.38 | 1.45 | 0.54 |

**Significant at p=0.01, *Significant at p=0.05
