# Supplementary material for: Optimization of ‘on farm’ hydropriming conditions in wheat: Soaking time and water volume have interactive effects on seed performance
Source: PLoS One. 2023 Jan 31;18(1):e0280962. doi: 10.1371/journal.pone.0280962 (PMC9888722; doi:10.1371/journal.pone.0280962)
Supplement: S9 Table — (DOCX) [file pone.0280962.s009.docx]

**S9 Table. Analysis of Variance (F-value) for effects of drying, temperature, water volume and soaking duration on germination characteristics, seedling growth and vigour indices of the wheat genotype WH-1124**

| **Source of variation** | **DF** | **Standard germination** | **Germination speed** | **Shoot length** | **Root length** | **Seedling length** | **Seedling fresh weight** | **Seedling dry weight** | **Seedling vigour**  **index-I** | **Seedling vigour**  **index-II** |
| --- | --- | --- | --- | --- | --- | --- | --- | --- | --- | --- |
| Drying | 1 | 4.39* | 1070.10** | 159.67** | 34.99** | 166.83** | 5.56* | 4.43* | 69.69** | 9.32** |
| Temperature | 1 | 5.23* | 224.37** | 4526.92** | 236.07** | 2605.64** | 556.14** | 153.07** | 591.29** | 49.95** |
| Water volume | 2 | 0.90 | 2.29 | 16.48** | 2.44 | 14.27** | 12.10** | 1.51** | 4.76* | 6.04** |
| Soaking duration | 3 | 9.24** | 320.88** | 327.33** | 27.08** | 220.40** | 182.02** | 27.83** | 96.92** | 33.25** |
| Temperature × Water volume | 2 | 0.23 | 1.69 | 3.98* | 0.05 | 1.47 | 2.74 | 0.35 | 0.19 | 0.43 |
| Temperature × Duration | 3 | 0.11 | 12.63** | 11.41** | 5.39** | 17.69** | 1.43 | 0.13 | 6.19** | 0.08 |
| Volume × Duration | 6 | 1.58 | 5.06** | 7.09** | 1.46 | 6.26** | 4.53** | 2.38* | 5.01** | 2.97* |
| Drying × Temperature | 1 | 0.00 | 15.52** | 14.62** | 25.58** | 56.24** | 0.13 | 2.55 | 16.25** | 1.42 |
| Drying × Water volume | 2 | 0.23 | 0.33 | 0.41 | 0.37 | 0.42 | 1.93 | 0.12 | 0.56 | 0.21 |
| Drying × Soaking duration | 3 | 0.50 | 116.58** | 18.00** | 4.73** | 19.14** | 2.07 | 1.83 | 7.94** | 1.83 |
| Temperature × Water volume × Soaking duration | 6 | 0.18 | 0.56 | 1.08 | 0.35 | 0.49 | 0.78 | 0.18 | 0.28 | 0.08 |
| Drying × Temperature × Water volume | 2 | 0.02 | 0.59 | 3.31 | 0.13 | 0.54 | 1.75 | 0.65 | 0.09 | 0.43 |
| Drying × Temperature × Soaking duration | 3 | 0.00 | 11.16** | 2.02 | 2.91* | 6.59** | 0.41 | 0.41 | 1.88 | 0.23 |
| Drying × Water volume × Soaking duration | 6 | 0.29 | 1.53 | 0.19 | 0.40 | 0.60 | 0.63 | 0.45 | 0.69 | 0.72 |
| Drying × Temperature × Water volume × Soaking duration | 6 | 0.03 | 0.74 | 0.57 | 0.08 | 0.23 | 0.40 | 0.32 | 0.10 | 0.18 |

**Significant at p=0.01, *Significant at p=0.05
