# Supplementary material for: Optimization of ‘on farm’ hydropriming conditions in wheat: Soaking time and water volume have interactive effects on seed performance
Source: PLoS One. 2023 Jan 31;18(1):e0280962. doi: 10.1371/journal.pone.0280962 (PMC9888722; doi:10.1371/journal.pone.0280962)
Supplement: S10 Table — (DOCX) [file pone.0280962.s010.docx]

**S10 Table. Analysis of Variance (F-value) for effects of drying, temperature, water volume and soaking duration on germination characteristics, seedling growth and vigour indices of the wheat genotype KRL-213**

| **Source of variation** | **DF** | **Standard germination** | **Germination speed** | **Shoot length** | **Root length** | **Seedling length** | **Seedling fresh weight** | **Seedling dry weight** | **Seedling vigour**  **index-I** | **Seedling vigour**  **index-II** |
| --- | --- | --- | --- | --- | --- | --- | --- | --- | --- | --- |
| Drying | 1 | 0.65 | 2080.03** | 23.59** | 1.71 | 19.58** | 2.40 | 5.02* | 12.40** | 5.98* |
| Temperature | 1 | 12.91** | 256.98** | 2474.97** | 352.77** | 2748.44** | 147.05** | 254.050** | 788.34** | 75.20** |
| Water volume | 2 | 1.05 | 27.06** | 18.37** | 7.28** | 25.40** | 7.79** | 12.70** | 15.05** | 11.33** |
| Soaking duration | 3 | 17.90** | 912.80** | 130.32** | 43.43** | 175.02** | 83.13** | 24.55** | 148.49** | 57.01** |
| Temperature × Water volume | 2 | 1.31 | 0.22 | 0.77 | 1.17 | 2.15 | 0.63 | 1.05 | 1.13 | 2.89 |
| Temperature × Duration | 3 | 0.21 | 21.34** | 6.60** | 1.37 | 7.40** | 2.10 | 0.84 | 4.06** | 1.10 |
| Volume × Duration | 6 | 1.51 | 9.78** | 3.66** | 1.91 | 5.75** | 3.15 | 1.58 | 6.22** | 2.75* |
| Drying × Temperature | 1 | 0.13 | 83.95** | 14.79** | 0.21 | 9.18** | 0.15 | 0.27 | 2.54 | 0.55 |
| Drying × Water volume | 2 | 1.05 | 7.48** | 1.91 | 1.18 | 0.99 | 0.10 | 1.43 | 1.99 | 0.02 |
| Drying × Soaking duration | 3 | 1.08 | 232.33** | 2.99* | 0.39 | 2.33 | 0.73 | 0.82 | 2.75* | 1.68 |
| Temperature × Water volume × Soaking duration | 6 | 0.42 | 0.05 | 0.43 | 0.20 | 0.53 | 1.44 | 0.41 | 0.41 | 0.65 |
| Drying × Temperature × Water volume | 2 | 0.60 | 0.54 | 0.14 | 0.04 | 0.16 | 0.03 | 0.41 | 0.73 | 1.39 |
| Drying × Temperature × Soaking duration | 3 | 0.16 | 13.12** | 1.71 | 0.09 | 1.11 | 0.25 | 0.17 | 0.46 | 0.33 |
| Drying × Water volume × Soaking duration | 6 | 0.26 | 0.95 | 0.62 | 0.28 | 0.67 | 0.40 | 0.19 | 0.90 | 0.17 |
| Drying × Temperature × Water volume × Soaking duration | 6 | 0.18 | 0.63 | 0.50 | 0.26 | 0.21 | 0.30 | 0.08 | 0.22 | 0.31 |

**Significant at p=0.01, *Significant at p=0.05
