# Supplementary material for: Optimization of ‘on farm’ hydropriming conditions in wheat: Soaking time and water volume have interactive effects on seed performance
Source: PLoS One. 2023 Jan 31;18(1):e0280962. doi: 10.1371/journal.pone.0280962 (PMC9888722; doi:10.1371/journal.pone.0280962)
Supplement: S11 Table — (DOCX) [file pone.0280962.s011.docx]

**S11 Table. Comparison between conventional and on-farm seed priming in case of the genotype WH 1105**

| **Drying method🠪** | **Surface dried (1 hour)**  **(On-Farm priming)** | | | | | | **Dried back^#^ (24 hours)**  **(Conventional priming)** | | | | | |
| --- | --- | --- | --- | --- | --- | --- | --- | --- | --- | --- | --- | --- |
| **Temperature🠪** | **20 °C** | | | **25 °C** | | | **20 °C** | | | **25 °C** | | |
| **Volume🠪**  **Soaking duration 🠇** | **Half** | **Equal** | **Double** | **Half** | **Equal** | **Double** | **Half** | **Equal** | **Double** | **Half** | **Equal** | **Double** |
|  | **Standard germination (%)** | | | | | | | | | | | |
| Control (unprimed) | 92.7 | 92.7 | 92.7 | 88.7 | 88.7 | 88.7 | 92.7 | 92.7 | 92.7 | 88.7 | 88.7 | 88.7 |
| 8 hours | 96.7 | 96.0 | 95.3 | 92.7 | 95.3 | 96.7 | 96.0 | 90.7 | 94.7 | 94.0 | 92.0 | 89.3 |
| 12 hours | 95.3 | 96.0 | 94.7 | 95.3 | 94.0 | 94.7 | 93.3 | 93.3 | 93.3 | 95.3 | 94.0 | 95.3 |
| 16 hours | 95.3 | 95.3 | 93.3 | 94.7 | 94.7 | 92.7 | 96.7 | 98.0 | 93.3 | 88.7 | 91.3 | 94.0 |
|  | **Speed of germination** | | | | | | | | | | | |
| Control (unprimed) | 47.3 | 47.3 | 47.3 | 48.7 | 48.7 | 48.7 | 47.3 | 47.3 | 47.3 | 48.7 | 48.7 | 48.7 |
| 8 hours | 62.7 | 64.4 | 67.1 | 79.3 | 86.0 | 72.4 | 53.1 | 53.3 | 51.4 | 57.1 | 56.0 | 60.0 |
| 12 hours | 76.4 | 80.7 | 72.7 | 80.4 | 85.8 | 82.0 | 53.3 | 54.2 | 52.9 | 57.8 | 55.8 | 54.4 |
| 16 hours | 79.3 | 78.4 | 69.8 | 81.3 | 82.0 | 80.2 | 53.3 | 58.9 | 47.8 | 56.7 | 58.7 | 50.2 |
|  | **Shoot length (cm)** | | | | | | | | | | | |
| Control (unprimed) | 10.5 | 10.5 | 10.5 | 12.9 | 12.9 | 12.9 | 10.5 | 10.5 | 10.5 | 12.9 | 12.9 | 12.9 |
| 8 hours | 11.1 | 11.1 | 11.0 | 14.5 | 14.9 | 14.3 | 10.4 | 10.6 | 10.9 | 13.2 | 12.2 | 12.9 |
| 12 hours | 12.4 | 11.8 | 11.8 | 15.8 | 15.7 | 14.5 | 11.8 | 11.2 | 11.7 | 14.1 | 14.1 | 13.9 |
| 16 hours | 12.3 | 11.8 | 11.7 | 15.7 | 15.4 | 15.0 | 11.5 | 11.8 | 11.8 | 13.7 | 13.0 | 12.7 |

***Continued…***

| **Drying method🠪** | **Surface dried (1 hour)**  **(On-Farm priming)** | | | | | | **Dried back^#^ (24 hours)**  **(Conventional priming)** | | | | | |
| --- | --- | --- | --- | --- | --- | --- | --- | --- | --- | --- | --- | --- |
| **Temperature🠪** | **20 °C** | | | **25 °C** | | | **20 °C** | | | **25 °C** | | |
| **Volume🠪**  **Soaking duration 🠇** | **Half** | **Equal** | **Double** | **Half** | **Equal** | **Double** | **Half** | **Equal** | **Double** | **Half** | **Equal** | **Double** |
|  | **Root length (cm)** | | | | | | | | | | | |
| Control (unprimed) | 18.9 | 18.9 | 18.9 | 19.6 | 19.6 | 19.6 | 18.9 | 18.9 | 18.9 | 19.6 | 19.6 | 19.6 |
| 8 hours | 19.0 | 19.7 | 19.3 | 21.5 | 22.0 | 21.7 | 19.4 | 19.5 | 18.9 | 18.9 | 18.9 | 19.5 |
| 12 hours | 20.1 | 19.8 | 19.4 | 21.5 | 22.4 | 20.6 | 20.4 | 19.9 | 17.9 | 21.6 | 20.7 | 20.7 |
| 16 hours | 19.7 | 19.6 | 19.2 | 21.1 | 21.3 | 18.8 | 19.6 | 19.5 | 19.0 | 20.0 | 21.1 | 21.1 |
|  | **Seedling length (cm)** | | | | | | | | | | | |
| Control (unprimed) | 29.4 | 29.4 | 29.4 | 32.5 | 32.5 | 32.5 | 29.4 | 29.4 | 29.4 | 32.5 | 32.5 | 32.5 |
| 8 hours | 30.2 | 30.8 | 30.3 | 35.9 | 36.9 | 36.0 | 29.9 | 30.1 | 29.8 | 32.1 | 31.1 | 32.4 |
| 12 hours | 32.6 | 31.6 | 31.1 | 37.4 | 38.0 | 35.1 | 32.1 | 31.1 | 29.7 | 35.7 | 34.8 | 34.6 |
| 16 hours | 32.1 | 31.4 | 30.9 | 36.8 | 36.7 | 33.8 | 31.1 | 31.3 | 30.8 | 33.7 | 34.1 | 33.8 |
|  | **Seedling fresh weight (mg)** | | | | | | | | | | | |
| Control (unprimed) | 152.0 | 152.0 | 152.0 | 176.0 | 176.0 | 176.0 | 152.0 | 152.0 | 152.0 | 176.0 | 176.0 | 176.0 |
| 8 hours | 163.0 | 181.0 | 174.0 | 186.3 | 185.0 | 185.7 | 157.0 | 165.0 | 164.7 | 180.7 | 169.7 | 181.3 |
| 12 hours | 188.7 | 190.3 | 173.0 | 202.3 | 211.7 | 190.3 | 135.0 | 154.0 | 166.0 | 196.0 | 189.7 | 196.7 |
| 16 hours | 180.0 | 170.3 | 179.3 | 202.0 | 210.3 | 188.3 | 190.7 | 173.3 | 187.3 | 201.7 | 193.0 | 194.3 |

***Continued…***

| **Drying method🠪** | **Surface dried (1 hour)**  **(On-Farm priming)** | | | | | | **Dried back^#^ (24 hours)**  **(Conventional priming)** | | | | | |
| --- | --- | --- | --- | --- | --- | --- | --- | --- | --- | --- | --- | --- |
| **Temperature🠪** | **20 °C** | | | **25 °C** | | | **20 °C** | | | **25 °C** | | |
| **Volume🠪**  **Soaking duration 🠇** | **Half** | **Equal** | **Double** | **Half** | **Equal** | **Double** | **Half** | **Equal** | **Double** | **Half** | **Equal** | **Double** |
|  | **Seedling dry weight (mg)** | | | | | | | | | | | |
| Control (unprimed) | 15.15 | 15.15 | 15.15 | 15.88 | 15.88 | 15.88 | 15.15 | 15.15 | 15.15 | 15.88 | 15.88 | 15.88 |
| 8 hours | 15.13 | 15.65 | 15.18 | 16.75 | 17.52 | 16.78 | 15.17 | 15.57 | 14.32 | 17.22 | 15.75 | 16.67 |
| 12 hours | 15.53 | 16.45 | 16.88 | 16.48 | 17.00 | 17.22 | 15.92 | 14.55 | 14.95 | 16.10 | 15.20 | 16.78 |
| 16 hours | 15.27 | 14.85 | 15.50 | 16.42 | 17.02 | 15.88 | 15.23 | 14.07 | 14.95 | 17.52 | 16.52 | 17.22 |
|  | **Seedling vigour index-I** | | | | | | | | | | | |
| Control (unprimed) | 2721 | 2721 | 2721 | 2876 | 2876 | 2876 | 2721 | 2721 | 2721 | 2876 | 2876 | 2876 |
| 8 hours | 2913 | 2958 | 2892 | 3329 | 3517 | 3480 | 2866 | 2723 | 2826 | 3017 | 2864 | 2895 |
| 12 hours | 3104 | 3033 | 2947 | 3562 | 3574 | 3320 | 3000 | 2899 | 2769 | 3398 | 3271 | 3293 |
| 16 hours | 3056 | 2994 | 2885 | 3482 | 3473 | 3136 | 3010 | 3069 | 2871 | 2990 | 3117 | 3173 |
|  | **Seedling vigour index-II** | | | | | | | | | | | |
| Control (unprimed) | 1403 | 1403 | 1403 | 1410 | 1410 | 1410 | 1343 | 1343 | 1343 | 1410 | 1410 | 1410 |
| 8 hours | 1463 | 1503 | 1448 | 1551 | 1670 | 1623 | 1426 | 1433 | 1280 | 1616 | 1449 | 1489 |
| 12 hours | 1480 | 1579 | 1598 | 1570 | 1599 | 1630 | 1518 | 1368 | 1426 | 1534 | 1427 | 1601 |
| 16 hours | 1457 | 1415 | 1447 | 1553 | 1610 | 1473 | 1352 | 1287 | 1404 | 1552 | 1510 | 1620 |

**#Dried back to original moisture content**
