# Supplementary material for: Optimization of ‘on farm’ hydropriming conditions in wheat: Soaking time and water volume have interactive effects on seed performance
Source: PLoS One. 2023 Jan 31;18(1):e0280962. doi: 10.1371/journal.pone.0280962 (PMC9888722; doi:10.1371/journal.pone.0280962)
Supplement: S12 Table — (DOCX) [file pone.0280962.s012.docx]

**S12 Table. Comparison between conventional and on-farm seed priming in case of the genotype WH 1124**

| **Drying method🠪** | **Surface dried (1 hour)**  **(On-Farm priming)** | | | | | | **Dried back^#^ (24 hours)**  **(Conventional priming)** | | | | | |
| --- | --- | --- | --- | --- | --- | --- | --- | --- | --- | --- | --- | --- |
| **Temperature🠪** | **20 °C** | | | **25 °C** | | | **20 °C** | | | **25 °C** | | |
| **Volume🠪**  **Soaking duration 🠇** | **Half** | **Equal** | **Double** | **Half** | **Equal** | **Double** | **Half** | **Equal** | **Double** | **Half** | **Equal** | **Double** |
|  | **Standard germination (%)** | | | | | | | | | | | |
| Control (unprimed) | 93.3 | 93.3 | 93.3 | 92.0 | 92.0 | 92.0 | 93.3 | 93.3 | 93.3 | 92.0 | 92.0 | 92.0 |
| 8 hours | 95.3 | 96.7 | 96.7 | 94.0 | 96.0 | 96.7 | 94.0 | 95.7 | 95.3 | 93.3 | 94.7 | 95.0 |
| 12 hours | 95.3 | 97.7 | 97.0 | 96.0 | 96.3 | 95.3 | 95.3 | 95.7 | 95.3 | 95.3 | 94.0 | 94.0 |
| 16 hours | 96.7 | 97.3 | 94.0 | 96.7 | 96.0 | 92.0 | 95.3 | 95.3 | 94.0 | 94.7 | 94.0 | 92.7 |
|  | **Speed of germination** | | | | | | | | | | | |
| Control (unprimed) | 50.4 | 50.4 | 50.4 | 54.4 | 54.4 | 54.4 | 50.4 | 50.4 | 50.4 | 54.4 | 54.4 | 54.4 |
| 8 hours | 65.8 | 63.8 | 67.3 | 80.0 | 84.0 | 84.4 | 55.8 | 56.0 | 55.1 | 58.7 | 60.4 | 62.7 |
| 12 hours | 73.3 | 78.7 | 76.4 | 80.7 | 85.6 | 82.9 | 57.8 | 56.9 | 54.9 | 61.3 | 60.9 | 63.8 |
| 16 hours | 77.8 | 77.8 | 70.7 | 83.1 | 80.9 | 77.8 | 57.3 | 57.8 | 53.1 | 61.1 | 63.1 | 58.9 |
|  | **Shoot length (cm)** | | | | | | | | | | | |
| Control (unprimed) | 10.2 | 10.2 | 10.2 | 13.1 | 13.1 | 13.1 | 10.2 | 10.2 | 10.2 | 13.1 | 13.1 | 13.1 |
| 8 hours | 11.1 | 11.9 | 11.7 | 15.0 | 15.7 | 16.1 | 10.5 | 11.3 | 11.6 | 14.0 | 14.6 | 14.8 |
| 12 hours | 12.0 | 12.1 | 11.8 | 15.3 | 15.8 | 16.1 | 11.0 | 11.6 | 11.5 | 14.4 | 14.9 | 14.8 |
| 16 hours | 12.5 | 12.4 | 11.8 | 15.6 | 15.9 | 16.0 | 11.6 | 11.5 | 11.4 | 14.5 | 14.9 | 14.8 |

***Continued…***

| **Drying method🠪** | **Surface dried (1 hour)**  **(On-Farm priming)** | | | | | | **Dried back^#^ (24 hours)**  **(Conventional priming)** | | | | | |
| --- | --- | --- | --- | --- | --- | --- | --- | --- | --- | --- | --- | --- |
| **Temperature🠪** | **20 °C** | | | **25 °C** | | | **20 °C** | | | **25 °C** | | |
| **Volume🠪**  **Soaking duration 🠇** | **Half** | **Equal** | **Double** | **Half** | **Equal** | **Double** | **Half** | **Equal** | **Double** | **Half** | **Equal** | **Double** |
|  | **Root length (cm)** | | | | | | | | | | | |
| Control (unprimed) | 19.1 | 19.1 | 19.1 | 20.1 | 20.1 | 20.1 | 19.1 | 19.1 | 19.1 | 20.1 | 20.1 | 20.1 |
| 8 hours | 19.4 | 19.8 | 20.0 | 21.9 | 22.1 | 23.0 | 19.3 | 19.5 | 19.5 | 20.2 | 20.4 | 20.9 |
| 12 hours | 19.6 | 20.1 | 20.2 | 22.2 | 23.0 | 22.9 | 19.6 | 19.9 | 19.9 | 20.8 | 21.0 | 21.3 |
| 16 hours | 19.6 | 20.0 | 19.6 | 22.3 | 22.9 | 21.7 | 19.7 | 19.9 | 19.8 | 21.1 | 21.1 | 21.0 |
|  | **Seedling length (cm)** | | | | | | | | | | | |
| Control (unprimed) | 29.3 | 29.3 | 29.3 | 33.1 | 33.1 | 33.1 | 29.3 | 29.3 | 29.3 | 33.1 | 33.1 | 33.1 |
| 8 hours | 30.5 | 31.7 | 31.7 | 36.8 | 37.8 | 39.0 | 29.8 | 30.8 | 31.1 | 34.2 | 35.0 | 35.7 |
| 12 hours | 31.7 | 32.2 | 32.0 | 37.5 | 38.8 | 39.1 | 30.6 | 31.4 | 31.4 | 35.2 | 35.9 | 36.0 |
| 16 hours | 32.1 | 32.4 | 31.3 | 38.0 | 38.8 | 37.6 | 31.3 | 31.4 | 31.2 | 35.6 | 36.0 | 35.8 |
|  | **Seedling fresh weight (mg)** | | | | | | | | | | | |
| Control (unprimed) | 155.7 | 155.7 | 155.7 | 181.3 | 181.3 | 181.3 | 155.7 | 155.7 | 155.7 | 181.3 | 181.3 | 181.3 |
| 8 hours | 162.3 | 179.7 | 181.7 | 193.0 | 197.0 | 198.3 | 163.3 | 173.3 | 173.3 | 189.3 | 188.3 | 200.7 |
| 12 hours | 184.0 | 198.0 | 193.7 | 207.7 | 215.3 | 213.7 | 175.0 | 189.7 | 187.3 | 205.0 | 206.0 | 214.3 |
| 16 hours | 181.0 | 186.3 | 177.7 | 210.7 | 218.0 | 201.0 | 179.3 | 188.3 | 181.0 | 206.0 | 210.3 | 210.3 |

***Continued…***

| **Drying method🠪** | **Surface dried (1 hour)**  **(On-Farm priming)** | | | | | | **Dried back^#^ (24 hours)**  **(Conventional priming)** | | | | | |
| --- | --- | --- | --- | --- | --- | --- | --- | --- | --- | --- | --- | --- |
| **Temperature🠪** | **20 °C** | | | **25 °C** | | | **20 °C** | | | **25 °C** | | |
| **Volume🠪**  **Soaking duration 🠇** | **Half** | **Equal** | **Double** | **Half** | **Equal** | **Double** | **Half** | **Equal** | **Double** | **Half** | **Equal** | **Double** |
|  | **Seedling dry weight (mg)** | | | | | | | | | | | |
| Control (unprimed) | 15.18 | 15.18 | 15.18 | 16.05 | 16.05 | 16.05 | 15.18 | 15.18 | 15.18 | 16.05 | 16.05 | 16.05 |
| 8 hours | 15.22 | 15.72 | 16.27 | 16.43 | 16.80 | 16.72 | 15.28 | 15.65 | 15.60 | 16.08 | 16.25 | 16.42 |
| 12 hours | 15.48 | 16.18 | 16.58 | 16.70 | 17.35 | 17.48 | 15.68 | 15.97 | 16.27 | 16.25 | 16.82 | 16.87 |
| 16 hours | 15.57 | 16.03 | 15.67 | 16.83 | 17.02 | 16.73 | 15.77 | 16.08 | 15.98 | 16.33 | 17.08 | 16.80 |
|  | **Seedling vigour index-I** | | | | | | | | | | | |
| Control (unprimed) | 2737 | 2737 | 2737 | 3047 | 3047 | 3047 | 2737 | 2737 | 2737 | 3047 | 3047 | 3047 |
| 8 hours | 2904 | 3063 | 3060 | 3463 | 3627 | 3773 | 2803 | 2947 | 2964 | 3189 | 3309 | 3392 |
| 12 hours | 3018 | 3144 | 3104 | 3603 | 3738 | 3725 | 2917 | 3008 | 2996 | 3352 | 3370 | 3385 |
| 16 hours | 3103 | 3152 | 2945 | 3669 | 3721 | 3462 | 2979 | 2994 | 2936 | 3366 | 3382 | 3321 |
|  | **Seedling vigour index-II** | | | | | | | | | | | |
| Control (unprimed) | 1417 | 1417 | 1417 | 1477 | 1477 | 1477 | 1417 | 1417 | 1417 | 1477 | 1477 | 1477 |
| 8 hours | 1451 | 1519 | 1573 | 1545 | 1613 | 1616 | 1437 | 1497 | 1487 | 1501 | 1538 | 1560 |
| 12 hours | 1476 | 1581 | 1608 | 1603 | 1671 | 1666 | 1496 | 1526 | 1551 | 1551 | 1581 | 1587 |
| 16 hours | 1505 | 1560 | 1472 | 1626 | 1633 | 1540 | 1503 | 1534 | 1503 | 1545 | 1606 | 1557 |

**#Dried back to original moisture content**
