# Supplementary material for: Optimization of ‘on farm’ hydropriming conditions in wheat: Soaking time and water volume have interactive effects on seed performance
Source: PLoS One. 2023 Jan 31;18(1):e0280962. doi: 10.1371/journal.pone.0280962 (PMC9888722; doi:10.1371/journal.pone.0280962)
Supplement: S13 Table — (DOCX) [file pone.0280962.s013.docx]

**S13 Table. Comparison between conventional and on-farm seed priming in case of the genotype KRL 213**

| **Drying method🠪** | **Surface dried (1 hour)**  **(On-Farm priming)** | | | | | | **Dried back^#^ (24 hours)**  **(Conventional priming)** | | | | | |
| --- | --- | --- | --- | --- | --- | --- | --- | --- | --- | --- | --- | --- |
| **Temperature🠪** | **20 °C** | | | **25 °C** | | | **20 °C** | | | **25 °C** | | |
| **Volume🠪**  **Soaking duration 🠇** | **Half** | **Equal** | **Double** | **Half** | **Equal** | **Double** | **Half** | **Equal** | **Double** | **Half** | **Equal** | **Double** |
|  | **Standard germination (%)** | | | | | | | | | | | |
| Control (unprimed) | 90.7 | 90.7 | 90.7 | 89.3 | 89.3 | 89.3 | 90.7 | 90.7 | 90.7 | 89.3 | 89.3 | 89.3 |
| 8 hours | 92.7 | 94.7 | 94.7 | 92.0 | 91.3 | 92.7 | 92.0 | 92.7 | 93.3 | 89.3 | 92.0 | 92.7 |
| 12 hours | 93.3 | 96.0 | 95.3 | 94.0 | 92.0 | 92.7 | 92.0 | 94.0 | 94.7 | 92.0 | 92.0 | 93.3 |
| 16 hours | 93.3 | 95.3 | 91.3 | 94.0 | 93.3 | 90.7 | 93.3 | 95.3 | 94.0 | 93.3 | 93.3 | 92.7 |
|  | **Speed of germination** | | | | | | | | | | | |
| Control (unprimed) | 44.4 | 44.4 | 44.4 | 45.3 | 45.3 | 45.3 | 44.4 | 44.4 | 44.4 | 45.3 | 45.3 | 45.3 |
| 8 hours | 65.8 | 67.3 | 68.9 | 78.9 | 82.9 | 83.1 | 49.6 | 52.9 | 56.9 | 51.8 | 54.9 | 58.4 |
| 12 hours | 70.9 | 75.1 | 71.8 | 81.1 | 85.6 | 83.6 | 49.8 | 55.6 | 56.4 | 53.6 | 59.8 | 59.1 |
| 16 hours | 72.2 | 76.4 | 69.3 | 82.4 | 84.4 | 79.1 | 52.7 | 56.0 | 54.7 | 54.7 | 61.6 | 56.9 |
|  | **Shoot length (cm)** | | | | | | | | | | | |
| Control (unprimed) | 10.73 | 10.73 | 10.73 | 13.33 | 13.33 | 13.33 | 10.73 | 10.73 | 10.73 | 13.33 | 13.33 | 13.33 |
| 8 hours | 11.28 | 11.74 | 11.93 | 14.47 | 15.12 | 15.62 | 11.23 | 11.48 | 11.58 | 14.02 | 14.15 | 14.67 |
| 12 hours | 11.27 | 12.18 | 12.11 | 14.73 | 15.58 | 15.83 | 11.63 | 11.78 | 12.00 | 14.20 | 14.93 | 15.05 |
| 16 hours | 11.68 | 12.10 | 12.02 | 15.37 | 16.02 | 15.38 | 11.85 | 11.98 | 12.02 | 14.68 | 14.87 | 15.12 |

***Continued…***

| **Drying method🠪** | **Surface dried (1 hour)**  **(On-Farm priming)** | | | | | | **Dried back^#^ (24 hours)**  **(Conventional priming)** | | | | | |
| --- | --- | --- | --- | --- | --- | --- | --- | --- | --- | --- | --- | --- |
| **Temperature🠪** | **20 °C** | | | **25 °C** | | | **20 °C** | | | **25 °C** | | |
| **Volume🠪**  **Soaking duration 🠇** | **Half** | **Equal** | **Double** | **Half** | **Equal** | **Double** | **Half** | **Equal** | **Double** | **Half** | **Equal** | **Double** |
|  | **Root length (cm)** | | | | | | | | | | | |
| Control (unprimed) | 18.1 | 18.1 | 18.1 | 19.2 | 19.2 | 19.2 | 18.1 | 18.1 | 18.1 | 19.2 | 19.2 | 19.2 |
| 8 hours | 18.3 | 18.4 | 19.0 | 19.7 | 19.8 | 20.3 | 18.2 | 18.5 | 18.7 | 19.4 | 19.6 | 20.5 |
| 12 hours | 18.9 | 19.1 | 19.0 | 20.3 | 20.6 | 21.0 | 18.6 | 18.9 | 19.1 | 19.9 | 20.2 | 20.8 |
| 16 hours | 19.1 | 19.0 | 18.7 | 20.5 | 20.5 | 20.5 | 18.8 | 18.8 | 19.1 | 20.1 | 20.4 | 20.6 |
|  | **Seedling length (cm)** | | | | | | | | | | | |
| Control (unprimed) | 28.8 | 28.8 | 28.8 | 32.6 | 32.6 | 32.6 | 28.8 | 28.8 | 28.8 | 32.6 | 32.6 | 32.6 |
| 8 hours | 29.6 | 30.2 | 30.9 | 34.2 | 34.9 | 35.9 | 29.5 | 30.0 | 30.3 | 33.4 | 33.8 | 35.2 |
| 12 hours | 30.2 | 31.2 | 31.1 | 35.0 | 36.2 | 36.8 | 30.2 | 30.7 | 31.1 | 34.1 | 35.1 | 35.8 |
| 16 hours | 30.8 | 31.1 | 30.7 | 35.8 | 36.6 | 35.9 | 30.6 | 30.8 | 31.1 | 34.8 | 35.3 | 35.8 |
|  | **Seedling fresh weight (mg)** | | | | | | | | | | | |
| Control (unprimed) | 146.0 | 146.0 | 146.0 | 163.5 | 163.5 | 163.5 | 146.0 | 146.0 | 146.0 | 163.5 | 163.5 | 163.5 |
| 8 hours | 158.2 | 155.7 | 159.5 | 171.5 | 179.5 | 187.5 | 155.5 | 154.5 | 163.0 | 167.1 | 175.3 | 189.0 |
| 12 hours | 170.0 | 176.0 | 181.5 | 173.5 | 195.5 | 189.5 | 163.0 | 169.8 | 174.0 | 176.7 | 188.0 | 183.0 |
| 16 hours | 169.0 | 180.5 | 178.5 | 189.0 | 190.3 | 185.8 | 171.5 | 177.2 | 169.7 | 187.5 | 192.2 | 185.0 |

***Continued…***

| **Drying method🠪** | **Surface dried (1 hour)**  **(On-Farm priming)** | | | | | | **Dried back^#^ (24 hours)**  **(Conventional priming)** | | | | | |
| --- | --- | --- | --- | --- | --- | --- | --- | --- | --- | --- | --- | --- |
| **Temperature🠪** | **20 °C** | | | **25 °C** | | | **20 °C** | | | **25 °C** | | |
| **Volume🠪**  **Soaking duration 🠇** | **Half** | **Equal** | **Double** | **Half** | **Equal** | **Double** | **Half** | **Equal** | **Double** | **Half** | **Equal** | **Double** |
|  | **Seedling dry weight (mg)** | | | | | | | | | | | |
| Control (unprimed) | 14.87 | 14.87 | 14.87 | 15.77 | 15.77 | 15.77 | 14.87 | 14.87 | 14.87 | 15.77 | 15.77 | 15.77 |
| 8 hours | 15.03 | 15.37 | 15.82 | 15.93 | 15.93 | 16.18 | 15.03 | 15.15 | 15.48 | 15.93 | 16.05 | 16.07 |
| 12 hours | 15.12 | 15.68 | 15.90 | 16.13 | 16.37 | 16.65 | 15.10 | 15.33 | 15.57 | 16.05 | 16.23 | 16.23 |
| 16 hours | 15.15 | 15.63 | 15.70 | 16.15 | 16.33 | 16.72 | 15.18 | 15.37 | 15.45 | 16.13 | 16.27 | 16.35 |
|  | **Seedling vigour index-I** | | | | | | | | | | | |
| Control (unprimed) | 2613 | 2613 | 2613 | 2909 | 2909 | 2909 | 2613 | 2613 | 2613 | 2909 | 2909 | 2909 |
| 8 hours | 2744 | 2854 | 2923 | 3147 | 3187 | 3327 | 2711 | 2780 | 2829 | 2987 | 3106 | 3263 |
| 12 hours | 2817 | 2998 | 2968 | 3288 | 3328 | 3410 | 2780 | 2884 | 2946 | 3139 | 3231 | 3342 |
| 16 hours | 2872 | 2966 | 2806 | 3367 | 3411 | 3252 | 2858 | 2938 | 2922 | 3243 | 3292 | 3313 |
|  | **Seedling vigour index-II** | | | | | | | | | | | |
| Control (unprimed) | 1347 | 1347 | 1347 | 1408 | 1408 | 1408 | 1347 | 1347 | 1347 | 1408 | 1408 | 1408 |
| 8 hours | 1393 | 1455 | 1497 | 1466 | 1455 | 1500 | 1383 | 1404 | 1445 | 1423 | 1478 | 1489 |
| 12 hours | 1411 | 1506 | 1516 | 1516 | 1507 | 1543 | 1389 | 1440 | 1473 | 1476 | 1494 | 1515 |
| 16 hours | 1414 | 1490 | 1434 | 1518 | 1524 | 1515 | 1417 | 1465 | 1452 | 1506 | 1518 | 1515 |

**#Dried back to original moisture content**
