# Supplementary material for: Optimization of ‘on farm’ hydropriming conditions in wheat: Soaking time and water volume have interactive effects on seed performance
Source: PLoS One. 2023 Jan 31;18(1):e0280962. doi: 10.1371/journal.pone.0280962 (PMC9888722; doi:10.1371/journal.pone.0280962)
Supplement: Attachment — Submitted filename: Point wise response to reviewer comments.docx [file pone.0280962.s014.docx]

**Point wise response to reviewer’s comments**

| **Editorial comments** | | |
| --- | --- | --- |
| **Sr. No.** | **Comment** | **Response** |
| 1. | The data analysis and writing of this paper are relatively good, and the analysis is relatively sufficient. | Authors are highly thankful to the learned reviewer for the comments. |
| 2. | In the part of materials and methods, I have a different opinion. Priming requires water absorption and dehydration. The dehydration process is also very important for priming. It can provide seeds with the ability to cope with water stress and other adversity. The author shortened the drying time to 1 hour. For the field sowing amount, one hour is not enough to dry seeds and is not convenience to sowing. I don't know why the author choose to dry for one hour after priming, not like other priming， drying to the initial moisture content. The paper has not designed a comparative experiment between conventional priming drying and 1-hour drying, which cannot prove that 1-hour drying is more effective than traditional priming. If the author supplements the experiment and the real data prove that his design is better than the traditional priming, I think the article can be published. | The reviewer has raised an important point.  We have conducted an experiment to compare the priming method described in the original manuscript (on-farm priming) and conventional priming (seeds dried to their original moisture content).  The relevant changes have been made in each section of the manuscript. In results section data is presented with help of bar graphs (Fig. 7-10). All the data generated in the experiment have been included in the supplementary section (Supp. Tables 8-13).  The results of the experiment revealed that on farm priming is better in terms of rapid germination and seedling growth and at par in case of germination percentage as compared to conventional priming. |
